# Dr. Andrew Wilson

**Published:** 1912-09-14

**Authors:** 


Dr. Andrew Wilson, of lay newspaper fame, lecturer on physiology and health to the George Combe Trust and Gilchrist Trust lecturer, has died suddenly at North Berwick. Born in Edinburgh in September 1852, he was educated at Edinburgh University. In 1876 he was appointed lecturer at the Edinburgh Medical School on zoology and comparative anatomy, and on these and kindred subjects he wrote a number of books and magazine articles.

